# Managing Metastatic Thymoma With Metabolic and Medical Therapy: A Case Report

**DOI:** 10.3389/fonc.2020.00578

**Published:** 2020-05-05

**Authors:** Matthew C. L. Phillips, Deborah K. J. Murtagh, Sanjay K. Sinha, Ben G. Moon

**Affiliations:** ^1^Department of Neurology, Waikato Hospital, Hamilton, New Zealand; ^2^Healthy Kitchen Christchurch Ltd., Hamilton, New Zealand; ^3^Department of Pathology, Waikato Hospital, Hamilton, New Zealand; ^4^Department of Radiology, Waikato Hospital, Hamilton, New Zealand

**Keywords:** metastatic thymoma, fasting, ketogenic diet, prednisone, cancer

## Abstract

Thymomas consist of neoplastic thymic cells intermixed with variable numbers of non-neoplastic lymphocytes. Metastatic thymomas are typically managed with non-curative chemotherapy to control tumor-related symptoms; no prolonged survival is expected. Metabolic-based approaches, such as fasting and ketogenic diets, target cancer cell metabolism by creating an increased reliance on ketones while decreasing glucose, glutamine, and growth factor availability, theoretically depriving cancer cells of their metabolic fuels while creating an unfavorable environment for cancer growth, which may be beneficial in metastatic thymoma. We report the case of a 37-year-old woman with myasthenia gravis, diagnosed with an inoperable type AB, stage IVA thymoma, who pursued a metabolic intervention consisting of periodic fasting (7-day, fluid-only fasts every 1–2 months), combined with a modified ketogenic diet on feeding days, for 2 years. Fasting-related adverse effects included cold intolerance, fatigue, and generalized muscle aches, all of which resolved during the second year. She experienced two myasthenia relapses, each associated with profoundly reduced oral intake, marked weight loss, and tumor regression-the first relapse was followed by a 32% decrease in tumor volume over 4 months, the second relapse by a dramatic 96% decrease in tumor volume over 4 months. The second relapse also required prednisone to control the myasthenia symptoms. We hypothesize that 2 years of fasting and ketogenic diet therapy metabolically weakened the neoplastic thymic cell component of the thymoma, “setting the stage” for immune activation and extreme energy restriction to destroy the majority of cancer cells during both relapses, while prednisone-induced apoptosis eradicated the remaining lymphocytic component of the thymoma during the second relapse. This case is unique in that a metabolic-based fasting and ketogenic diet intervention was used as the primary management strategy for a metastatic cancer in the absence of surgery, chemotherapy, or radiotherapy, culminating in a near-complete regression. Nearly 3 years after being diagnosed with inoperable metastatic cancer, our patient shows no signs of disease and leads a full and active life.

## Introduction

Thymomas, the most common tumor of the anterior mediastinum, are composed of neoplastic thymic epithelial cells intermixed with variable numbers of non-neoplastic lymphocytes (1, 2). Thymomas are usually asymptomatic but may present with chest pain, dyspnea, and a variety of autoimmune disorders, most commonly myasthenia gravis which presents with ocular, bulbar, and limb weakness and fatiguability (3). Nearly 30% of thymomas are inoperable (4), resulting in 5-year survival rates of 36–53% (5, 6). Inoperable, metastatic thymomas are typically managed with chemotherapy to control tumor-related symptoms; no prolonged survival is expected (4). Novel therapeutic strategies are needed for metastatic thymomas.

Cancer is generally regarded as a primarily genetic disorder, yet it may also be perceived as a primarily metabolic disorder, with most of the genetic abnormalities arising as secondary phenomena (7). Cancer cells show a dramatically increased uptake of glucose, a feature common to over 90% of malignant cancers (8), and some cancer cells also show increased uptake of the amino acid glutamine (9, 10). Cancer cells rely upon these fermentable metabolites to compensate for mitochondria dysfunction and impaired cell respiration, which are characteristic of most cancers (7). Cancer cells also rely heavily on growth signaling pathways, particularly those involving insulin, insulin-like growth factor-1 (IGF-1), and mammalian target of rapamycin (mTOR) (11), to support a “reprogrammed” cell metabolism redirected toward unbridled growth and proliferation (12). Given these facts, cancer cells may be vulnerable to interventions that selectively target their abnormal metabolism.

Metabolic interventions, such as fasting and ketogenic diets, target cancer cell metabolism and may be effective alongside medical therapies in treating advanced cancers (7, 13). Fasting is a voluntary abstinence from food and drink for specified, recurring periods of time, with the fasting periods typically ranging from 12 h to 3 weeks in humans, whereas ketogenic diets are high-fat, adequate-protein, low-carbohydrate diets that stimulate the body to mimic a fasted metabolic state (14). Both interventions increase fat metabolism within the body, the former utilizing endogenous (body) fat and the latter exogenous (dietary) fat. After several days of fasting or a ketogenic diet, the human body enters a state of physiological ketosis characterized by low blood glucose levels, emptied liver glycogen stores, and hepatic production of ketones, which serve as a major energy source for brain and muscle. Ketones cannot be effectively utilized by cancer cells and may inhibit their growth (15, 16). Moreover, both interventions can decrease glucose, glutamine, and growth factor availability, depriving cancer cells of their major fuels and creating an unfavorable physiological environment for unchecked growth and proliferation. To our knowledge, neither fasting nor ketogenic diets have been utilized as the primary management strategy for metastatic cancer in the absence of surgery, chemotherapy, or radiotherapy.

## Case Report

We report the case of a 37-year-old, 37 weeks pregnant marketing consultant who presented with 2 months of eyelid weakness worsening on activity as well as 1 month of pleuritic chest pain and dyspnea. She was diagnosed with myasthenia gravis at age 26, during which time a coexisting mediastinal mass was laparoscopically resected and diagnosed as a World Health Organization (WHO) type B2 invasive thymoma. Although she had been intermittently managed with pyridostigmine, prednisone, and intravenous immunoglobulin (IVIg) in the interim, her only current medication was azathioprine 50 mg orally daily. She weighed 53 kg. A neurological examination revealed subtle bilateral asymmetric ptosis and mild eyelid fatiguability. Examination findings regarding upper and lower limb power, reflexes, plantar responses, and sensation were normal.

**Figure 1:**
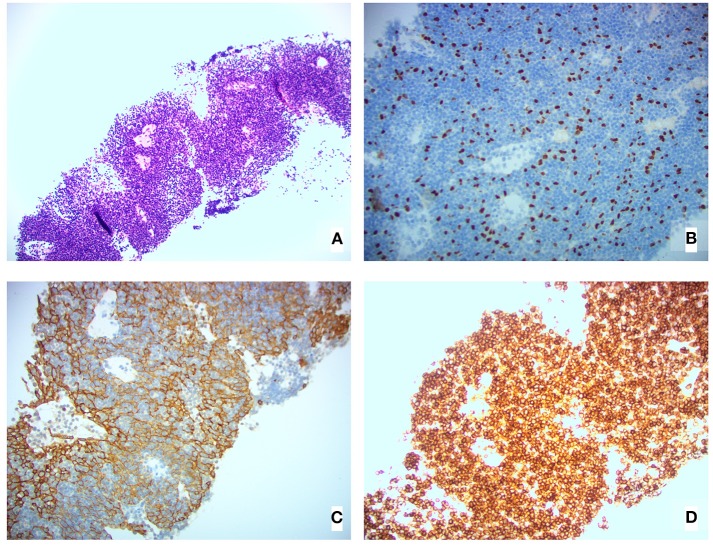
Pleural biopsy histological images showing **(A)** H&E stain, **(B)** 20×P63 stain (highlighting epithelial cell nuclei), **(C)** 20×AE1/AE3 stain (highlighting epithelial cell cytoplasm), and **(D)** 20×CD45 stain (highlighting lymphoid cells).

A computed tomography (CT) scan of the neck and chest revealed several large soft tissue masses in the left lung, the largest being 10 × 5 × 14 cm (total tumor volume 549.6 cm^3^); several masses invaded the left pleura, and there was a small left pleural effusion. Our patient was treated with IVIg (1 g/kg, administered over 5 days) to ensure the uncomplicated delivery of a healthy baby girl 2 weeks later. She underwent a left pleural percutaneous needle biopsy the day after delivery and was diagnosed with a WHO type AB (indicating oval/spindle cells admixed with abundant small lymphocytes), Masaoka stage IVA (indicating pleural or pericardial dissemination) thymoma [Figure 1; (3)]. The thymoma was deemed unresectable at a multidisciplinary meeting, and she was offered non-curative chemotherapy, which she declined. Following this, a combined metabolic intervention was offered; after all foreseeable risks and benefits had been explained, she chose this course.

**Figure 2:**
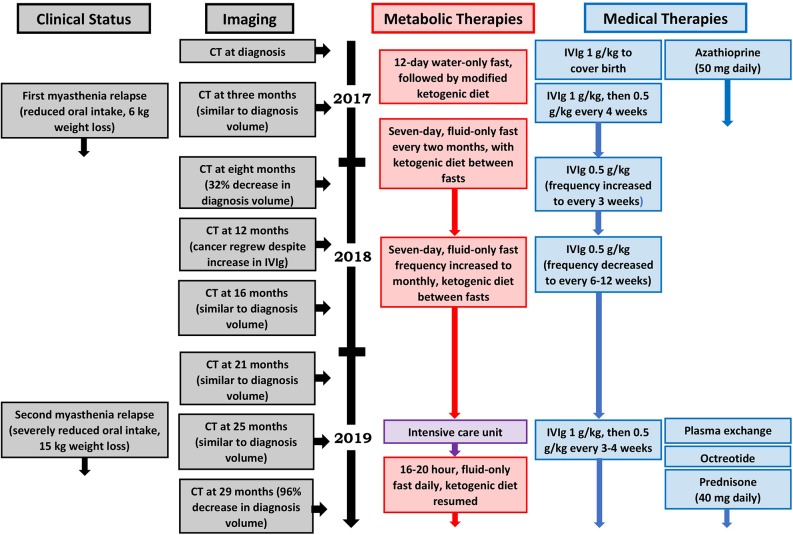
Patient timeline.

**Figure 3:**
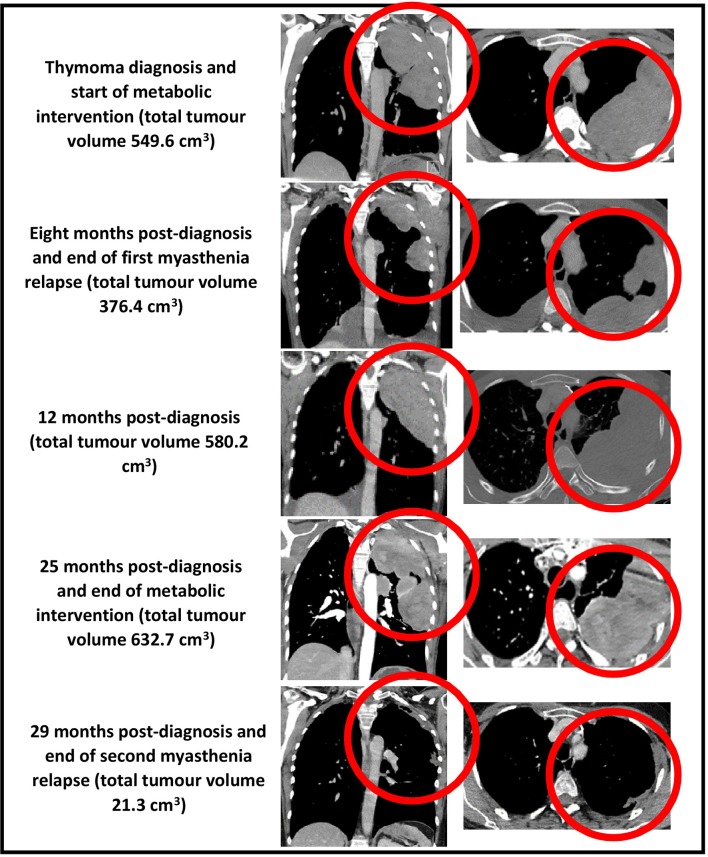
CT chest (coronal and axial views, bulk of tumor circled in red) showing total tumor volumes at diagnosis, 8, 12, 25, and 29 months post-diagnosis.

Our patient's health and myasthenia gravis were regularly monitored by a neurologist during the metabolic intervention, which consisted of a periodic fasting regimen (starting with a 12-day, water-only fast followed by a series of 7-day, fluid-only fasts every 1–2 months) combined with a modified ketogenic diet (60% fat, 30% protein, 5% fiber, and 5% net carbohydrate by weight, consisting largely of green vegetables, meats, eggs, nuts, seeds, creams, and natural oils) on feeding days (Figure 2). She monitored and recorded her blood glucose and beta-hydroxybutyrate (BHB) levels (Freestyle Neo; Abbott Diabetes Care, Whitney, UK) three times per week (17). All adverse effects were documented. An oncologist monitored the thymoma with a CT scan every 4–5 months, and a radiologist blinded to treatment simultaneously assessed all CT scans with volumetric analysis at the end of the intervention (Figure 3). Tumor volumes were calculated by Hounsfield unit threshold segmentation followed by manual correction of all tumor margins.

The first 3 months of the combined metabolic intervention was uneventful, but during months 4–7, our patient experienced reduced appetite and oral intake in the setting of several weeks of diarrhea (up to 10 motions per day), resulting in 6-kg weight loss; the diarrhea was extensively investigated and thought to be a thymoma-associated autoimmune enteropathy (18). Coincident with the diarrhea, she developed a myasthenia relapse resulting in worsening bilateral ptosis, dysphagia, and four-limb weakness with fatiguability. Our patient chose not to commence prednisone as she had experienced disrupted mood, insomnia, and weight gain several years previously while taking this medication. She was therefore commenced on 4-weekly IVIg for 4 months, after which her 8-month CT scan revealed a partial regression of the tumor (376.4 cm^3^, representing a 32% decrease in diagnosis volume), moderate bilateral pleural effusions, and a small pericardial effusion. Given that the myasthenia symptoms persisted combined with the possibility that the IVIg may have contributed to the partial regression, the azathioprine was stopped and the IVIg frequency increased to 3-weekly.

During months 9–12, our patient's diarrhea ceased, she regained her weight, and the myasthenia symptoms resolved. However, the 12-month CT demonstrated that the thymoma had regrown to its original diagnosis volume. Given her ongoing myasthenia control and lack of IVIg inhibitory effect on the growth of the tumor, the IVIg frequency was decreased to 6-weekly.

During months 13–24, our patient remained largely free of diarrhea and maintained her diagnosis body weight, and the myasthenia symptoms remained controlled. Her IVIg frequency was decreased to 8-weekly and then 12-weekly. By the time of the 25-month CT, the thymoma showed minimal growth (632.7 cm^3^, representing a 13% increase in diagnosis volume), and all effusions had disappeared.

After 2 years on the combined fasting and ketogenic diet intervention, our patient weighed 54 kg and her mean 2-year blood glucose and BHB levels (±standard deviation) were measured at 4.98 ± 0.55 and 3.50 ± 1.27 mmol/L, respectively. During each 7-day fasting period, she lost an average of 2.9 ± 0.72 kg of body weight, with mean blood glucose and BHB levels during the fasting periods measured at 3.92 ± 0.73 and 6.31 ± 1.55 mmol/L, respectively. There were several fasting-related adverse effects including cold intolerance, fatigue, and generalized muscle aches, all of which peaked during the first 3 days of each fasting period and progressively resolved over the first year such that they no longer occurred by the second year. No adverse effects occurred in relation to the ketogenic diet.

During months 25–29, our patient experienced several weeks of drastically reduced oral intake and diarrhea, resulting in 15-kg weight loss. She also developed a second, more severe myasthenia relapse resulting in bilateral ptosis, dysarthria, four-limb weakness with fatiguability, and respiratory failure requiring 2 weeks of intubation and mechanical ventilation in the intensive care unit. She was treated with plasma exchange followed by 3- to 4-weekly IVIg and 10 days of octreotide 1.5 mg subcutaneously daily (followed by a single dose of long-acting octreotide 20 mg intramuscularly 1 month later). Despite the previous adverse effects experienced by our patient in relation to prednisone, we decided to commence prednisone 40 mg orally daily. Her myasthenia symptoms resolved, and the 29-month CT revealed a near-complete regression of the thymoma (21.3 cm^3^, representing a 96% decrease in diagnosis volume).

## Discussion

In this case, a metabolic-based fasting and ketogenic diet intervention, along with adjunctive medications aimed at controlling myasthenia symptoms, culminated in the near-complete regression of a metastatic thymoma. For 2 years, our patient relied almost completely upon a metabolic strategy to manage her metastatic cancer, during which time she remained active, maintained her diagnosis body weight, and the tumor volume increased by a modest 13%. There were several fasting-related adverse effects including cold intolerance, fatigue, and generalized muscle aches, all of which resolved by the second year. She then experienced a 4-month myasthenia relapse, accompanied by severe weight loss and requiring prednisone, during which time the thymoma decreased in volume by 96%. Nearly 3 years after being diagnosed with metastatic cancer, our patient shows no signs of disease and leads a full and active life. She continues her metabolic therapy and her only remaining medical therapy is prednisone 10 mg orally daily.

Various metabolic approaches are theoretically capable of targeting cancer cell energy metabolism by creating an increased cell reliance on ketone and fat metabolism, a decreased reliance on glucose as the primary metabolic fuel, and reduced levels of tumor growth-promoting factors such as insulin, IGF-1, and mTOR (11, 14). Normal cells are metabolically flexible and readily adapt to ketone and fat metabolism; in contrast, metabolically inflexible cancer cells undergo physiological stress (19). The metabolic intervention supported by the most evidence is calorie restriction, defined as a chronic 20–40% reduction in calorie intake with maintained meal frequency (20). Calorie restriction reduces tumor incidence by 75% in rodents and by 50% in rhesus monkeys (21, 22). However, long-term adherence to calorie restriction is challenging in cancer patients (11); from a practical standpoint, fasting and ketogenic diets are more suitable. Periodic fasting (fasting periods lasting 2 days or longer) holds a particular therapeutic edge by inducing more extreme changes in ketone, glucose, glutamine, and growth factor levels compared to calorie restriction or ketogenic diets, as shown by the lower glucose and higher BHB levels measured by our patient during the fasts. Although the benefits of fasting interventions in preventing cancer in animals are somewhat variable (21), they can exceed those of calorie restriction (23, 24); there is also mounting evidence that fasting may benefit human cancer patients, particularly when combined with conventional treatments such as chemotherapy (25–27). Regarding ketogenic diets, many animal studies suggest an antitumor effect; however, evidence of improved outcomes in human cancer patients is currently limited to individual cases (28).

It is important to note that although 2 years of a combined fasting and ketogenic diet intervention may have limited the growth of our patient's metastatic thymoma, the tumor had not decreased in volume by the end of this approach; it was only in the setting of two myasthenia relapses that significant volume reduction occurred, with a 32% decrease in tumor diagnosis volume during the first 4-month relapse and a 96% decrease in volume during the second 4-month relapse. Given that both relapses in our patient were characterized by abnormal immune function and marked weight loss, it is possible that immune activation and extreme energy restriction contributed to the regressions, and since both relapses were treated medically, one or more of the myasthenia medications may have contributed to the regressions. These possibilities warrant discussion.

First, it is possible that immune activation contributed to both regressions. So-called “spontaneous” regressions have been documented for thousands of years in a variety of cancers (29). The mechanism of spontaneous regression remains unknown but may involve the activation of antigen recognition mechanisms such that the immune system becomes capable of recognizing cancer cells, allowing for the establishment of active immunity against tumors (30). In the case of thymomas, which are generally asymptomatic, the few documented cases of spontaneous regression presented with fever, chest pain, and pleural effusions which are thought to result from a massive inflammatory reaction within the tumor (31). Given the similar findings in this case, an immunity-induced regression may have occurred in our patient, although it must be noted that the spontaneous regression of a metastatic thymoma in the absence of any other treating factors is exceedingly rare; to our knowledge, only one case of a stage IVA thymoma undergoing a spontaneous (and only partial) regression has been reported (31).

Second, extreme energy restriction may have contributed to both regressions. During each relapse, our patient experienced a profound reduction in appetite resulting in minimal calorie intake over several weeks. The ensuing weight loss was considerable—for example, during each 1-week, fluid-only fast, our patient typically lost 2.9 kg (5% of body weight), whereas during the first and second relapses, she lost 6 kg (11% of body weight), and 15 kg (28% of body weight), respectively. In both cases, such a degree of weight loss would have created drastic alterations in ketone, glucose, glutamine, and growth factor levels, fostering a hostile physiological environment for metabolically inflexible cancer cells. Thus, it is possible that an extreme metabolic-induced regression occurred in our patient.

Third, one or more of the medications used to treat our patient's myasthenia symptoms may have contributed to the regressions. The possibility that IVIg contributed to the first regression was considered and partially formed the rationale for increasing the IVIg frequency from 4- to 3-weekly in week 9. Despite this adjustment, the thymoma regrew to its original size by week 12, which suggests that IVIg did not contribute to either regression. In the second and more pronounced regression, octreotide and prednisone were additionally used to placate the myasthenia gravis and autoimmune enteropathy. Octreotide inhibits somatostatin receptors, dampening angiogenesis and growth factor availability, which can produce objective response rates of 10–37% in thymic tumors (32, 33). However, due to funding restrictions, octreotide was only administered for 10 days in our patient, followed by a single long-acting dose 1 month later; it seems unlikely that so small a dose could significantly contribute to the dramatic reduction in thymoma volume that occurred. Although extremely rare, corticosteroid-induced regressions of advanced thymomas in the absence of other treatments have been documented (34–36); however, all cases involved subtype B1 thymomas which contain more CD4+ and CD8+ double-positive immature lymphocytes compared to other subtypes (37). Double-positive lymphocytes show a high expression of glucocorticoid receptors, rendering the lymphocytic component of subtype B1 thymomas susceptible to glucocorticoid-induced apoptosis, whereas neoplastic thymic cells and hence other subtypes (including type AB, as in this case) are resistant to this mechanism (37). Nonetheless, it remains possible that prednisone-induced lymphocytic apoptosis contributed to the second regression seen in our patient, although it cannot explain the first regression.

Taken together, it is likely that several factors culminated in the near-complete regression of our patient's metastatic thymoma. It is difficult to ignore 2 years of periodic fasting combined with a ketogenic diet, which would have immersed the tumor in ketones while depriving it of glucose, glutamine, and growth-promoting factors. It is equally difficult to ignore the two myasthenia relapses in which immune activation and extreme energy restriction may have contributed to both regressions; in the case of the second relapse, it is possible that prednisone-induced apoptosis of the lymphocytic component of the tumor contributed to the dramatic second regression. Overall, we hypothesize that 2 years of a combined fasting and ketogenic diet intervention metabolically weakened the neoplastic thymic cell component of the thymoma, “setting the stage” for immune activation and extreme energy restriction to destroy the majority of cancer cells during the relapses, with prednisone-induced apoptosis destroying most of the lymphocytic component of the thymoma during the second relapse, culminating in the virtual eradication of the tumor.

Given that this study involved one patient, its major limitation is obvious, and it is difficult to draw definitive conclusions. Additional potential limitations include concerns that periodic fasting and ketogenic diets may produce unwanted weight loss and other adverse effects in patients with metastatic cancer. It is therefore important to note that our patient did not experience weight loss after 2 years undergoing both metabolic interventions. Furthermore, although she experienced several fasting-related adverse effects, these were all transient and improved as she adapted to each successive fasting period.

In conclusion, this case is unique in that a metabolic-based fasting and ketogenic diet intervention was used as the primary management strategy for a metastatic cancer in the absence of surgery, chemotherapy, or radiotherapy, culminating in the near-complete regression of an inoperable metastatic thymoma, with our patient experiencing only transient, fasting-related side effects. Nearly 3 years after being diagnosed with inoperable metastatic cancer, our patient shows no signs of disease and leads a full and active life. Although we cannot be certain of the mechanism underlying this remarkable outcome, the most plausible explanation is that 2 years of fasting and ketogenic diet therapy metabolically weakened the thymoma, setting the stage for a combined immunity-induced, metabolic-induced, and prednisone-induced near-complete regression. Despite our uncertainty, the extraordinary outcome in our patient highlights the importance of exploring metabolic-based therapies in advanced cancer cases, in the hope that more options may be offered to patients in the years to come.

## Ethics Statement

Written informed consent was obtained from the patient in this case report for the publication of any potentially identifiable images or data included in this article.

## Author Contributions

MP: conception, design, interpretation, and write-up of final article. DM: diet implementation and advice, and proof-reading of final article. SS: histology analysis and advice, and proof-reading of final article. BM: imaging analysis and advice, and proof-reading of final article.

## Conflict of Interest

DM was employed by the company Healthy Kitchen Christchurch Ltd. The remaining authors declare that the research was conducted in the absence of any commercial or financial relationships that could be construed as a potential conflict of interest.
